# Hybrid Nanoparticles of Citrate-Coated Manganese Ferrite and Gold Nanorods in Magneto-Optical Imaging and Thermal Therapy

**DOI:** 10.3390/nano13030434

**Published:** 2023-01-20

**Authors:** Saeideh Arsalani, Soudabeh Arsalani, Mileni Isikawa, Eder J. Guidelli, Ernesto E. Mazon, Ana Paula Ramos, Andris Bakuzis, Theo Z. Pavan, Oswaldo Baffa, Antonio A. O. Carneiro

**Affiliations:** 1 Department of Physics, FFCLRP, University of São Paulo, Av. Bandeirantes 3900, Ribeirão Preto 14040-901, São Paulo, Brazil; 2 Physikalisch-Technische Bundesanstalt, Abbestrasse 2-12, D-10587 Berlin, Germany; 3 Department of Chemistry, FFCLRP, University of São Paulo, Av. Bandeirantes 3900, Ribeirão Preto 14040-901, São Paulo, Brazil; 4 Institute of Physics and CNanoMed, Federal University of Goiás, Goiânia 74690-900, São Paulo, Brazil

**Keywords:** Ci-MnFe_2_O_4__CTAB-GNRs hybrid NPs, contrast agent, magneto-motive ultrasound imaging, magnetic hyperthermia, photoacoustic imaging

## Abstract

The development of nanomaterials has drawn considerable attention in nanomedicine to advance cancer diagnosis and treatment over the last decades. Gold nanorods (GNRs) and magnetic nanoparticles (MNPs) have been known as commonly used nanostructures in biomedical applications due to their attractive optical properties and superparamagnetic (SP) behaviors, respectively. In this study, we proposed a simple combination of plasmonic and SP properties into hybrid NPs of citrate-coated manganese ferrite (Ci-MnFe_2_O_4_) and cetyltrimethylammonium bromide-coated GNRs (CTAB-GNRs). In this regard, two different samples were prepared: the first was composed of Ci-MnFe_2_O_4_ (0.4 wt%), and the second contained hybrid NPs of Ci-MnFe_2_O_4_ (0.4 wt%) and CTAB-GNRs (0.04 wt%). Characterization measurements such as UV-Visible spectroscopy and transmission electron microscopy (TEM) revealed electrostatic interactions caused by the opposing surface charges of hybrid NPs, which resulted in the formation of small nanoclusters. The performance of the two samples was investigated using magneto-motive ultrasound imaging (MMUS). The sample containing Ci-MnFe_2_O_4__CTAB-GNRs demonstrated a displacement nearly two-fold greater than just using Ci-MnFe_2_O_4_; therefore, enhancing MMUS image contrast. Furthermore, the preliminary potential of these hybrid NPs was also examined in magnetic hyperthermia (MH) and photoacoustic imaging (PAI) modalities. Lastly, these hybrid NPs demonstrated high stability and an absence of aggregation in water and phosphate buffer solution (PBS) medium. Thus, Ci-MnFe_2_O_4__CTAB-GNRs hybrid NPs can be considered as a potential contrast agent in MMUS and PAI and a heat generator in MH.

## 1. Introduction

Nanomaterials have been widely exploited in biomedical applications over the past few decades [1,2,3]. As an example, gold nanorods (GNRs) are known as promising candidates due to their biocompatibility [4], being well-defined in terms of size and having tunable localized surface plasmon resonance [5]. As a result of these striking properties, GNRs have attracted significant interest in optical imaging and therapeutic techniques such as photoacoustic imaging (PAI) and photothermal therapy (PTT) [4,6,7,8].

In addition, magnetic nanoparticles (MNPs) have been extensively used in a variety of applications such as biology and biomedicine, owing to their unique features [9,10,11,12,13,14,15,16,17]. Manganese ferrites (MnFe_2_O_4_) are interesting spinel ferrite NPs among the various mixed ferrites (Afe_2_O_4_) with other transition metal ions (e.g., A = Mn, Ni, Cu, and Zn) due to their biocompatibility, saturation magnetization, and chemical stability [18,19,20,21,22,23]. Considering the advantages of MnFe_2_O_4_, these NPs are promising candidates to develop theranostic platforms, particularly in the field of personalized nanomedicine like magnetic resonance imaging (MRI) [24,25] and magnetic hyperthermia (MH) [26,27,28].

Several studies have combined the benefits of iron oxide nanoparticles (IONPs) and GNRs in magnetic and optical/thermal imaging modalities [29,30,31,32]. For example, in PAI, endogenous chromophores in tissue such as melanin and hemoglobin can generate a noticeable signal; therefore, reducing the sensitivity of PAI to identify the region marked with the plasmonic NPs. The integration of PAI and MMUS have been proposed to overcome this barrier by combining magneto-plasmonic NPs [29,30,31,32,33]. Qu et al. created liposomes encapsulating IONPs (Fe_3_O_4_) and GNRs as a dual-contrast agent for magneto-photoacoustic imaging to improve contrast in both ex vivo and in vivo studies [29,31,33]. Furthermore, the same research group [34] suggested using nanoclusters containing gold nanospheres and IONPs in MMUS imaging to improve the accuracy of effective PTT. More specifically, MMUS imaging can assess tissue elasticity, which has been identified as a critical parameter in PTT efficiency evaluation [34]. Although several studies have been conducted using such hybrid NPs as magneto-plasmonic NPs in biomedical applications, contrast enhancement using citrate-coated manganese ferrite (Ci-MnFe_2_O_4_) and cetyltrimethylammonium-bromide-coated GNRs (CTAB-GNRs) in MMUS has not been previously reported.

In the current study, we suggest a hybrid NP, for theranostic purpose, made of Ci-MnFe_2_O_4_ and CTAB-GNRs through a simple combination in which CTAB-GNRs and Ci-MnFe_2_O_4_ were synthesized using a gold-seed-mediated method [35] and a coprecipitation route [27], respectively. The interaction of these NPs was investigated using several characterizations, including a magnetic separation system (SEPMAG), UV-Visible spectroscopy, transmission electron microscopy (TEM), and attenuated total reflection (ATR). To highlight their stability in physiological media, the colloidal stability of hybrid NPs in phosphate buffer solution (PBS) at pH 7.4 (physiological pH) was also studied. Moreover, the performance of using only Ci-MnFe_2_O_4_ and its combination with CTAB-GNRs was investigated in MMUS, as well as their preliminary potential in the PAI and MH.

## 2. Experimental Section

### 2.1. Materials

The chemical reagents used were: cetyltrimethylammonium bromide (CTAB), sodium borohydride (NaBH_4_), tetrachloroauric acid (HauCl_4_·4H_2_O), silver nitrate (AgNO_3_), and L-ascorbic acid (AA), which were purchased from Sigma Aldrich (Burlington, MA, USA), Vetec (Rio de Janeiro, Brazil), Sigma Aldrich (Burlington, MA, USA), Cennabras (São Paulo, Brazil) and Panreac (Barcelona, Spain), respectively. Milli-Q water was also used for the preparation and washing solutions.

### 2.2. Methods

#### 2.2.1. Preparation of Ci-MnFe_2_O_4_ NPs

Ci-MnFe_2_O_4_ NPs were synthesized via the coprecipitation route by Zufelato et al. [27]. The core, crystal, hydrodynamic sizes, and polydispersity index (PDI) of these MNPs were 16.7, 14, 38 nm, and 0.32, respectively. In addition, Ci-MnFe_2_O_4_ had a saturation magnetization of 52.54 emu/g (in powder). For further information about MNPs synthesis and characterization, the reader is referred to the Supplementary Material (Figures S1a–c and S2).

#### 2.2.2. Preparation of CTAB-GNRs

GNRs coated by CTAB were manufactured in two steps according to the study of Morasso et al. [35]: Preparation of gold seed NPs and growth solution.

##### Gold Seed NPs

Gold seeds were synthesized according to the study by Morasso et al. [35] with only one modification by replacing hydroquinone (acting as a reducing agent) with AA. First, a 5 mL of HAuCl_4_ solution of 0.5 mM was added to the CTAB solution (200 mM) at 40 °C. Next, 0.6 mL of 10 mM fresh ice-cold NaBH_4_ was added under vigorous stirring. The mixture’s color changed rapidly to light brown, confirming the formation of small gold NPs [5,35]. The stirring was continued for 20 more minutes. A schematic illustration of the gold seed preparation is shown in Figure S3a.

##### Preparation of Growth Solution

In the next step (the growth solution), we added 55 μL of AA, 200 μL of AgNO_3_ solution (4 mM), and 5 mL of HauCl_4_ solution (1 mM), respectively, to 80 mM of CTAB under vigorous stirring. Thereafter, 12 μL seed suspension was added to the growth solution [35], as shown in Figure S3b. The mixture was then stirred for 60 min. The color of the solutions changed to light ruby after 20 min, indicating the formation of GNRs.

##### Purification of CTAB-GNRs

As mentioned earlier, the GNRs were stabilized by CTAB; therefore, the suspension containing CTAB-GNRs was centrifuged to remove any excess of CTAB due to its cytotoxicity. The GNRs were precipitated at the bottom of the solvent after 8 min of centrifugation at 7000 rpm (Eppendorf 5415D Microcentrifuge with Rotor F45-24-11). The GNRs were then resuspended in Milli-Q water, depending on the amount of residue. Finally, the GNR suspension was kept at room temperature.

#### 2.2.3. Preparation of Ci-MnFe_2_O_4__CTAB-GNRs Hybrid NPs

The concentrations of the stock dispersion of CTAB-GNRs and Ci-MnFe_2_O_4_ were 0.35 wt% and 3 wt%, respectively. A hybrid NP dispersion with lower concentration was papered for both MMUS and MH experiments, such that it consisted of 0.04 wt% CTAB-GNRs and 0.4% Ci-MnFe_2_O_4_ NPs. The MMUS experiments were conducted using samples of 900 μL volume; that is, 103 μL of CTAB-GNRs and 120 μL of Ci-MnFe_2_O_4_ were taken from their corresponding stock and dispersed in solution of 6 wt% gelatin to reach the final volume of 900 μL. For MH experiments, the samples were prepared by dispersing 66 μL Ci-MnFe_2_O_4_ and 57 μL CTAB-GNRs of the stocks in water to reach the final volume of 500 μL (the same concentration as in MMUS samples). Prior to the experiments, these hybrid NPs were mixed using a 3D rotation mixer for 24 h to allow their interactions to occur.

### 2.3. Characterization of NPs

Various techniques were used to characterize NPs. Spectrophotometric analysis, in the visible and near-infrared regions, was used to determine the optical properties that provide information about the size of the gold seeds and GNRs via the plasmonic band and to investigate the interaction of Ci-MnFe_2_O_4__CTAB-GNRs. The experiments were conducted using a UV spectrometer (Ultrospec 2100 pro, Amersham Pharmacia Biotech, Amersham, UK) with a resolution of 0.5 nm operating in the wavelength range of 200–900 nm. Furthermore, TEM measurements were performed on a JEOL-JEM-100 CXII (Peabody, MA, USA) to verify the structure and size of CTAB-GNRs and Ci-MnFe_2_O_4_, as well as to confirm the electrostatic interaction of hybrid NPs made of Ci-MnFe_2_O_4_ and CTAB-GNRs. A droplet of the desired suspension was dried on the copper grid at room temperature for TEM samples. The ImageJ software was used to calculate the mean diameters from TEM images (more than 200 particles per sample were counted). The Origin^®^ software 2019b (OriginLab, Northampton, MA, USA) was then used to plot the histograms of NPs dimensions obtained from the TEM images.

XRD (D5005 Diffractometer, Bruker-AXS, São Paulo, Brazil) analysis was used to determine the crystalline properties and phase identification, with X-ray beam nickel-filtered copper K radiation (=1.5406) in the range 10° < 2θ° < 70°. Next, the hydrodynamic diameter, PDI, and Zeta potential of NPs were determined by dynamic light scattering (DLS) and using a Zetasizer Nano ZS (Malvern Instrument, Malvern, UK). The data were measured at a fixed angle (173°) and an Nd: YAG laser (532 nm). Following that, an attenuated total reflectance (ATR) accessory coupled to a Fourier-transform infrared spectrophotometer (FTIR) was used to investigate CTAB molecule binding on the surface of GNRs, the functionalization surface of MnFe_2_O_4_ coated with sodium citrate, and particle interaction.

Next, the magnetic properties of Ci-MnFe_2_O_4_ and its combination with CTAB-GNRs were investigated by a vibrating sample magnetometer (VSM, EG&G Princeton Applied Research Magnetometer, São Paulo, Brazil) at room temperature using powder samples. Then, a magnetic separation system (SEPMAG, Barcelona, Spain) was used to verify the interactions between Ci-MnFe_2_O_4_ and CTAB-GNRs. More details about magnetic separation measurement are described in the following section.

#### Magnetic Separation

A magnetic separation system was used to measure the separation time of the aforementioned NPs, which highly depends on particle size distribution [10,36]. In this study, we used a magnetic separation system to examine the interaction between Ci-MnFe_2_O_4_ and CTAB-GNRs by measuring the separation time of Ci-MnFe_2_O_4_ and Ci-MnFe_2_O_4__CTAB-GNRs samples separately.

This system is based on the movement of MNPs under the influence of magnetic field gradients. This phenomenon is known as magnetophoresis, which is defined by the magnetophoretic velocities of the MNPs as a result of the separation time parameter [37,38]. The equipment contains two small cylindrical cavities with a volume of 2 mL and a third with a larger volume (15 mL). In this device, a homogeneous magnetic gradient of 15 T/m was applied by permanent magnets to create uniform magnetophoretic conditions for the three cavities. The magnetic force acting on magnetic particles can be defined as follows [39,40]:

(1) $$F=m\mu_{0}\frac{\partial H}{\partial r}$$

in which *μ*_0_ is the vacuum magnetic permeability constant, $\frac{\partial H}{\partial r}$ is the radial component of the magnetic gradient, and *m* is the magnetic moment of the particle, which is expressed as follows:

(2) $$m=M_{s}\rho_{\rho }\frac{4}{3}\pi R^{3}$$

*M_S_*, *ρ_ρ_*, and *R* are the saturation magnetization per unit mass of the colloid, the particle density, and the particle hydrodynamic radius, respectively. There is also the drag force opposing the magnetic field motion, which is given by [39]:

(3) $$F_{d}=6\pi \eta Rv$$

in which *η* is the viscosity of the fluid and *v* is the velocity of the particles. Therefore, the particles move toward the walls with a magnetophoretic velocity determined by the balance of the forces in Equations (1) and (3) [41,42]:

(4) $$\nu =\frac{2M_{s}\rho_{\rho }R^{2}}{9\eta }$$

**Figure 1:**
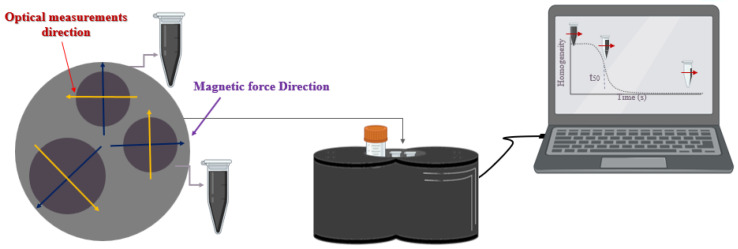
A schematic top view of the magnetic separation setup, which has three cavities with a volume of 2 mL for two tubes and 15 mL for the third tube. The red arrows indicate the movement of MNPs under the influence of magnetic field gradients.

It should be noted that this system includes an optical sensor for measuring the transmitted light, which is produced by an LED array. The opacity of the sample changes over time during the process [36]. To be more precise, the maximum opacity is observed at the beginning of the process (*t*_0_) due to the solution’s homogeneity. Half separation time (*t*_50_), which is the time when the opacity decreases by 50%, is employed to examine the magnetophoretic behavior of the samples. A schematic illustration of the magnetic separation process is shown in Figure 1.

### 2.4. Gelatin Tissue-Mimicking Phantom

Gelatin/agar tissue-mimicking phantoms were prepared to perform the MMUS and PAI experiments. This preparation consisted of two steps. First, the inclusion was prepared using a hemispherical mold (1 cm in diameter). To do so, 6 wt% gelatin (GELITA, São Paulo, Brazil) was dissolved in deionized water at 25 °C and heated to 70 °C to obtain a homogeneous solution. When the temperature reached 70 °C, the solution was kept at room temperature and slowly mixed to cool down to 40 °C; formaldehyde was then added considering 5 wt% of the gelatin’s mass [43]. Finally, the phantom was placed in the refrigerator for 24 h [3]. Three different inclusions were manufactured as follows: the first inclusion was made of only 0.40 wt% Ci-MnFe_2_O_4_, the second was prepared using hybrid NPs of 0.40 wt% Ci-MnFe_2_O_4__0.04 wt% CTAB-GNRs, and the last sample was also made of hybrid NPs with the concentration of GNRs increased to 0.07 wt%. Since this study mainly focused on magnetic applications, low concentrations of CTAB-GNRs (0.04 and 0.07 wt%) were utilized to investigate their impact after mixing with 0.40 wt% Ci-MnFe_2_O_4_ on the MMUS contrast.

The next step was to assemble the background of the phantoms with a cylindrical mold (7 cm in diameter and 2.5 cm in height). This part of phantom was made using the same procedures as previously described for inclusion preparation [10], but with a single modification of mixing 6 wt% gelatin with 3 wt% agar (HIMEDIA supplied Bacteriologic CAT. RM026, Thane, India). In this case, the solution was heated to 90 °C to achieve a uniform mixture. Three samples of each phantom type were created for a total of six phantoms.

### 2.5. MMUS Experimental Setup

The MMUS experimental setup consisted of a coil with 130 turns, an inner diameter of 22 mm, 114.2 µH of inductance, and 217.9 mΩ of DC resistance. A steel core of 20 mm diameter with a coercivity of 20 A/m was inserted in the center of the coil to enhance and focus the magnetic field. The tip of the steel core was positioned 2 mm away from the phantom’s central region. The system also included a half-drive inverter to charge the capacitor bank once it reached the desired voltage. After charging the capacitor, an electronic switching device and the coil generated the magnetic field pulse. For further information about the MMUS setup, please refer to Mazon et al. [44]. A multichannel ultrasound pulse/echo system (Sonix RP + Sonix DAQ, Ultrasonix) was then used to track the induced displacement of the internal structure (in the order of micrometers) by a cross-correlation method [45]. It should be mentioned that the US acquisition was synchronized with the magnetic excitation through a computer using a LabVIEW interface. This system operated with a frame rate of 4 kHz, and the magnetic pulse duration varied from 4 to 8 ms [44]. The maximum magnetic field applied 2 mm from the tip of the core was 740 mT. A schematic of the pulsed MMUS setup is shown in Figure S4.

### 2.6. PAI Setup

The PAI measurements were carried out using an Nd: YAG laser (Brilliant B, Quantel, Les Ulis, France) coupled to an optical parametric oscillator (MagicPRISM, Opotek, Carlsbad, CA, USA). The optical beam was delivered to the phantom via a trifurcated optical fiber bundle (Oriel Instrument, Newport, RI, USA) attached to a linear L14-5/38 ultrasound transducer (Ultrasonix Medical Corp., Richmond, BC, Canada). A parallel acquisition module (SonixDAQ, Ultrasonix, Richmond, BC, Canada) was used to collect PA data [46]. GNRs are commonly used as photo-absorbers in PAI due to their excellent optical absorption property; the first phantom was made using only a low concentration of CTAB-GNRs (0.04 wt%). The second and third phantoms contained 0.4 wt% Ci-MnFe_2_O_4_ and hybrid NPs of 0.4 wt% Ci-MnFe_2_O_4_ 0.04 wt% CTAB-GNRs, respectively, similar to those used for MMUS. Thus, the potential of hybrid NPs for PAI was examined. For each phantom, 49 frames were acquired and averaged to obtain the PA images using the optical wavelength of 750 nm, corresponding to the longitudinal absorption peak of the CTAB-GNRs. The laser energy level was recorded to compensate for pulse-to-pulse variation, and the beam mean energy at the phantom surface was 10.30 ± 0.37 mJ, 10.23 ± 0.38 mJ, and 9.71 ± 0.39 mJ, for phantoms 1, 2, and 3, respectively.

### 2.7. MH Experiments

This experiment was conducted using a homemade MH system [47]. The applied magnetic field had a sinusoidal and continuous profile with amplitude of 10 mT at 132 kHz. Three samples containing Ci-MnFe_2_O_4_ (0.4 wt%) and the hybrid NPs of Ci-MnFe_2_O_4_ (0.4 wt%)_CTAB-GNRs (0.04 wt% and 0.07 wt%) were dispersed in Milli-Q water and positioned on a holder inside a solenoid. The diameter and height of this solenoid are 14 and 87 mm, respectively, and it can generate a homogeneous magnetic field across the entire sample volume. A fiber optic thermometer system (Qualitrol NOMAD-Touch Fiber Optic Monitor, QC, Canada) was used to record the temperature of the samples [48,49,50]. Moreover, the power dissipated and converted into heat by both samples was calculated using the specific loss power (SLP) expression as shown below [48]:

(5) $$SLP=\frac{C_{w}m_{w}}{m_{np}}\frac{\Delta T}{\Delta t}$$

The Box–Lucas equation was used to fit the results of temperature versus time, according to the reference [49,51] in which: *C_np_* is the volume-specific heat capacity of the sample, *m_np_* is the MNPs’ mass, *m_w_* is the mass of the dispersion (which is water), and *C_w_* is the specific heat capacity of water. In addition, the intrinsic loss power (ILP) was also calculated to provide a better comparison with the SLP values reported in other studies [49].

## 3. Results and Discussion

UV-Vis/near-infrared measurements of gold seeds were conducted to confirm the formation of gold seeds (Figure S5a). Their size should be small (around 5 nm) to ensure that gold seed NPs could be used in the following procedure (growth solution). As a result, no plasmonic peak was expected to be observed in the range of 500 to 520 nm (Figure S5a). In addition, the TEM image showed the generation of gold seeds with spherical morphology and a size of about 5 ± 1 nm (see Figure S5b), which agrees with the literature [5,35].

**Figure 2:**
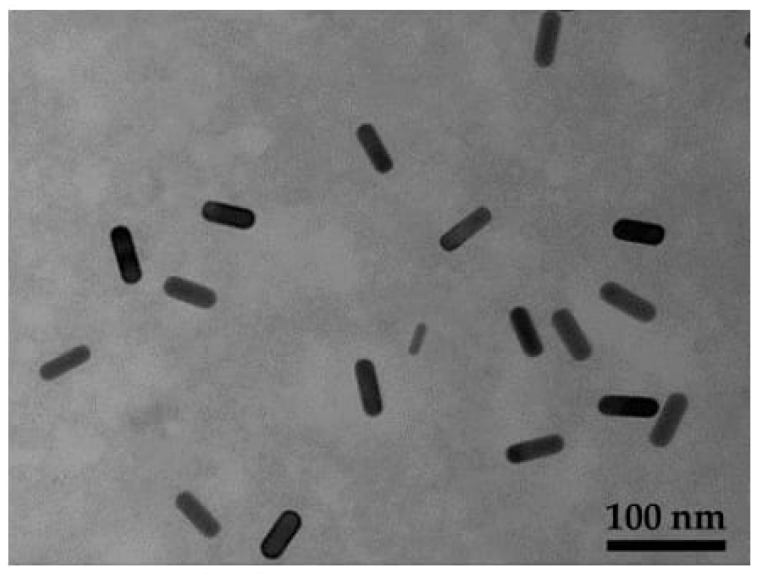
TEM images of CTAB-GNRs in the scale bar of 100 nm.

Furthermore, the UV-Visible spectra of CTAB-GNRs revealed transverse and longitudinal plasmon bands at 515 and 744 nm, respectively, providing information about the size and shape of GNRs (Figure S6a). The TEM image of GNRs depicted that they are rod-shaped and uniform in size, as shown in Figure 2. The average length and width of CTAB-GNRs were 42.3 ± 4.1 nm and 15.31 ± 1.5 nm, respectively, with an aspect ratio of 2.76 (Figure S6b,c).

In this study, hybrid NPs containing 0.4 wt% Ci-MnFe_2_O_4_ and 0.04 wt% CTAB-GNRs were thoroughly investigated. However, as another example, partial results of 0.4 wt% Ci-MnFe_2_O_4_ and 0.07 wt% CTAB-GNRs are presented here, such as hydrodynamic size, Zeta potential, MMUS, and MH.

**Figure 3:**
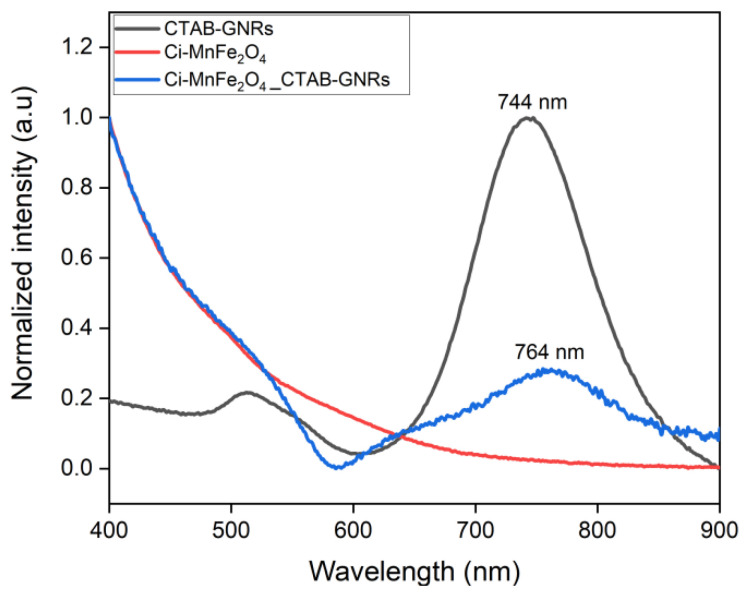
UV-Vis-NIR absorbance spectra of the solutions include CTAB-GNRs (black), Ci-MnFe_2_O_4_ (red), and Ci-MnFe_2_O_4__CTAB-GNRs hybrid NPs (blue).

The UV-Visible measurement was then carried out to confirm the interactions between CTAB-GNRs and Ci-MnFe_2_O_4_. The normalized optical absorbance spectra of the suspensions containing CTAB-GNRs, Ci-MnFe_2_O_4_, and hybrid NPs are shown in Figure 3.

The longitudinal peak of CTAB-GNRs can be seen at 744 nm, while the plasmonic band of CTAB-GNRs showed a redshift to 764 nm after mixing with Ci-MnFe_2_O_4_. The band at 764 nm is related to the CTAB-GNRs, revealing a redshift of the plasmonic band, which has a broader peak upon interaction with the Ci-MnFe_2_O_4_. Other studies have reported similar results [29,31]. Furthermore, a small absorption peak at around 650 nm was observed for hybrid NPs (blue curve), which could be due to the formation of clusters, which decreases the extinction coefficient because of the presence of larger particles. As a result, the plasmonic intensity of the dipole mode decreases, making the plasmonic band of the GNRs with smaller aspect ratios more noticeable, which was previously embedded/hidden by the high-intensity longer wavelength dipolar plasmon band [52,53,54].

**Figure 4:**
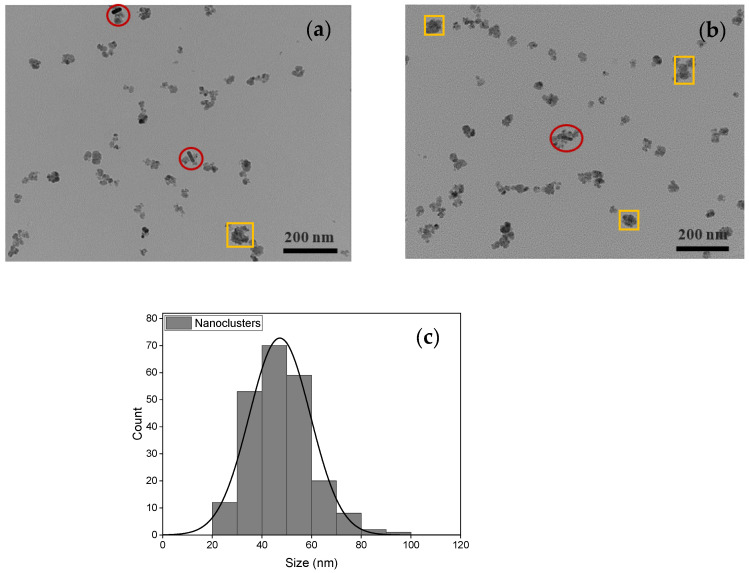
(**a**,**b**) TEM images and (**c**) histogram of nanoclusters of Ci-MnFe_2_O_4_ (yellow rectangular) and 0.4 wt% Ci-MnFe_2_O_4__0.04 wt% CTAB-GNRs hybrid NPs (red circles). The scale bar corresponds to 200 nm.

Figure 4a,b show TEM images of the interaction between Ci-MnFe_2_O_4__CTAB-GNRs hybrid NPs (red circles). Since the GNRs coated with CTAB had a positive surface charge, and the manganese ferrite stabilized by a capping agent of citrate had a negative surface charge, it was expected to generate an electrostatic attraction between these NPs (red circles). These results agree with the study by Truby [55], which showed excellent decoration of TREG SPIONs (positive charge) around the surface of the GNRs (negative charge) owing to charge affinity. In addition, small nanoclusters of Ci-MnFe_2_O_4_ were formed (yellow rectangular) after adding CTAB-GNRs to Ci-MnFe_2_O_4_ due to a charge imbalance in the medium. As expected, only a few CTAB-GNRs are observed compared to Ci-MnFe_2_O_4_ in the TEM images of hybrid NPs (Figure 4a,b). The reason could be the low amount of CTAB-GNRs used (0.04 wt%), while the concentration of Ci-MnFe_2_O_4_ used was much higher (nearly ten times greater (0.4 wt%) than CTAB-GNRs) in this study. Thus, more Ci-MnFe_2_O_4_ compared to CTAB-GNRs was expected to be observed in TEM images. The average particle size of nanoclusters was estimated to be around 48 ± 12 nm, as shown in Figure 4c.

Zeta potential was used to analyze the stability of the employed NPs (Table 1). GNRs coated with CTAB and MnFe_2_O_4_ capped with sodium citrate demonstrated a Zeta potential of +41 mV and −43.5 mV, respectively, indicating that the NPs surfaces were adequately coated and produced stable colloids, as shown in Table 1. After combining different concentrations of CTAB-GNRs with Ci-MnFe_2_O_4_, they maintained good stability at −30.4 mV and −31.1 mV. Following the classical colloidal theory, suspension stability can be interpreted as the balance between repulsive forces (with electrostatic origin) and attractive forces (generally associated with van der Waals interactions) [56]. The Zeta potential values found for individual NPs (i.e., +41 mV and −43.5, respectively, CTAB-GNRs and Ci-MnFe_2_O_4_) correlate with a sufficient repulsive force to attain better physical colloidal stability. When these two particles interact, the net charge of the hybrid NPs decreases, and the electrostatic repulsion weakens [57]. This condition favors attractive forces to dominate the interaction between individual NPs of the hybrid NPs, reducing the electrostatic stability (Zeta potential = −30.4 mV and −31.1 mV for 0.4 wt% CiMnFe_2_O_4__0.04 wt% CTAB-GNRs and 0.4 wt% CiMnFe_2_O_4__0.07 wt% CTAB-GNRs, respectively). Furthermore, the stability of hybrid NPs of 0.4 wt% Ci-MnFe_2_O_4__0.04 wt% CTAB-GNRs was repeated after 6 months, and it maintained its stability (−31.6 mV) with no sedimentation. The colloidal stability of both hybrid NPs dispersed in PBS at pH 7.4 (physiological pH) was also investigated [58]. Surprisingly, in PBS buffer with pH 7.4, these hybrid NPs showed high stability (Table 1), and after immersion in PBS medium, their average hydrodynamic sizes did not change. It should be noted that the minor difference in hydrodynamic size and PDI of hybrid NPs dispersed in water or buffer is most likely due to a difference in the concentration used, as DLS analysis is highly concentration dependent. In addition, slightly higher PDI values after immersion in PBS could be attributed to the lack of ultrasonication for NPs prior to DLS measurements. As a result, these hybrid NPs could maintain their dispersion stability and absence of aggregation in physiological conditions.

Some studies have suggested that the GNRs can be coated with polyethylene glycol (PEG), polystyrene sulfonate (PSS), and polyallylamine hydrochloride (PAH) to improve the stability and overcome cytotoxicity of CTAB [8,55,59]. Meanwhile, other factors such as size and concentration influence on the toxicity of GNRs and should be considered [8]. Our results reinforce the relevance of physical characterizations using physical phantoms for this kind of NP prior to addressing safety and reliability issues before in vivo assays.

**Figure 5:**
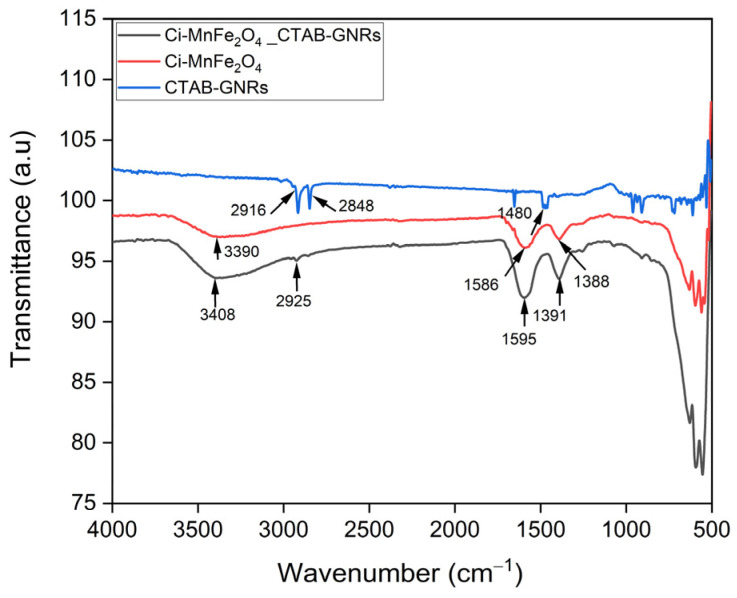
FTIR spectra of CTAB-GNRs, Ci-MnFe_2_O_4_, and their combination. The arrows indicate the main features of the spectra.

The ATR-FTIR spectra of CTAB-GNRs (blue line) confirmed the adsorption of the surfactant at the surface of the NP (Figure 5) due to the presence of bands at 2848 and 2916 cm^−1^ assigned to the C–H symmetric and antisymmetric stretching. The less intense band at 1480 cm^−1^ is related to the amine group of the quaternarium ammonium salt. Moreover, the region of 961 and 910 cm^−1^ can be related to the presence of N(CH_3_)_2_ group. The FTIR spectrum of Ci-MnFe_2_O_4_ (red line) exhibits the presence of bands at 1388 cm^−1^ and 1586 cm^−1^, which are assigned to the symmetric and antisymmetric stretchings of C-O, respectively. The broad band related to the vibration of -OH at 3390 cm^−1^ also confirmed the existence of adsorbed citrate molecules on the MnFe_2_O_4_ surface. The displacement of the OH- and C-O-related bands in the FTIR spectrum of Ci-MnFe_2_O_4__CTAB-GNRs (black line) to a lower/higher wavenumber suggests the interaction between the two NPs by hydrogen bonding [60], assisted by the presence of CTAB and citrate on the surfaces.

**Figure 6:**
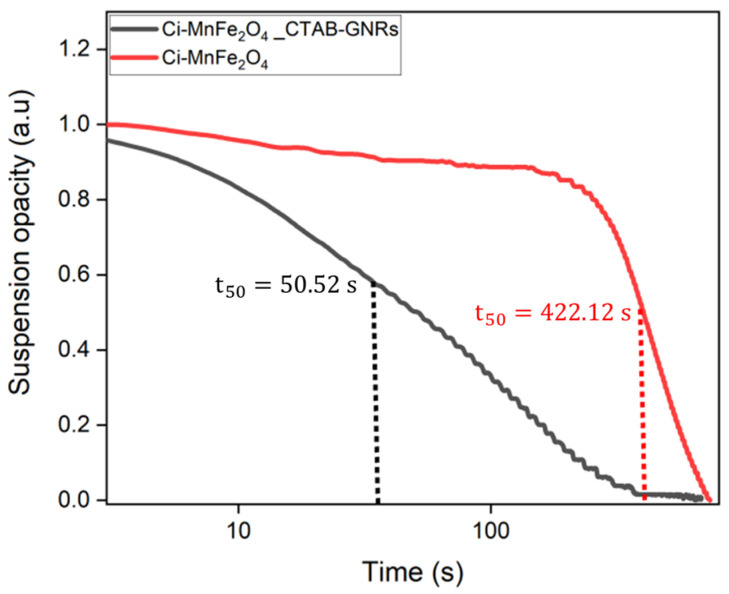
The magnetophoretic curve of Ci-MnFe_2_O_4_ and its combination with CTAB-GNRs.

Furthermore, the magnetophoretic behavior of both samples was studied. Based on the obtained results, the separation time of Ci-MnFe_2_O_4_ (422.12 s) considering an intermediate stage (*t*_50_) was significantly longer than that of Ci-MnFe_2_O_4__CTAB-GNRs hybrid NPs (50.52 s), as shown in Figure 6, indicating the presence of larger NPs or clustering in the environment. According to Equation (1), the attractive magnetic force rises as the size of the hybrid NPs increases due to the presence of nanoclusters compared to Ci-MnFe_2_O_4_. Thus, hybrid NPs in the solution moved faster toward the tube wall (Equation (4)), resulting in a shorter separation time. These results can confirm the interactions between Ci-MnFe_2_O_4_ and CTAB-GNRs and the presence of larger hydrodynamic particle sizes. Our results agree with the study of Leonie Wittmann et al. [61], who investigated the effect of MNP movement along a magnetic field gradient on hydrodynamic particle size and found that larger NPs had a quicker separation time.

**Figure 7:**
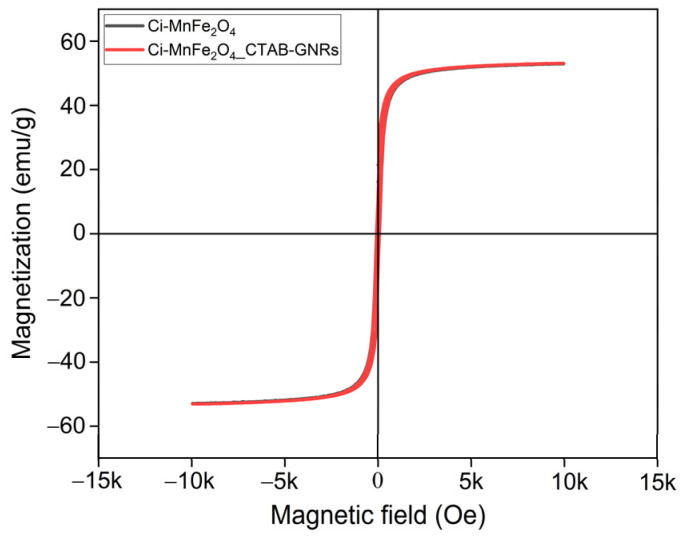
Magnetization curves of Ci-MnFe_2_O_4_ and its combination with CTAB-GNRs were recorded by a VSM, considering the total mass of each sample.

Figure 7 shows the M-H curves of Ci-MnFe_2_O_4_ and its combination with CTAB-GNRs in the applied field of −10 to +10 kOe at room temperature, considering the total mass. The magnetization of both samples exhibits superparamagnetic behavior. The saturation magnetization for both samples (MnFe_2_O_4_ with and without CTAB-GNRs) was almost the same at 52.54 emu/g and 52.8 emu/g, respectively. Since the CTAB-GNRs concentration was too low (0.04 wt.% for the hybrid NPs), there was no effect on the magnetization results, and both samples reported similar magnetization saturation in a high field (10 kOe).

**Figure 8:**
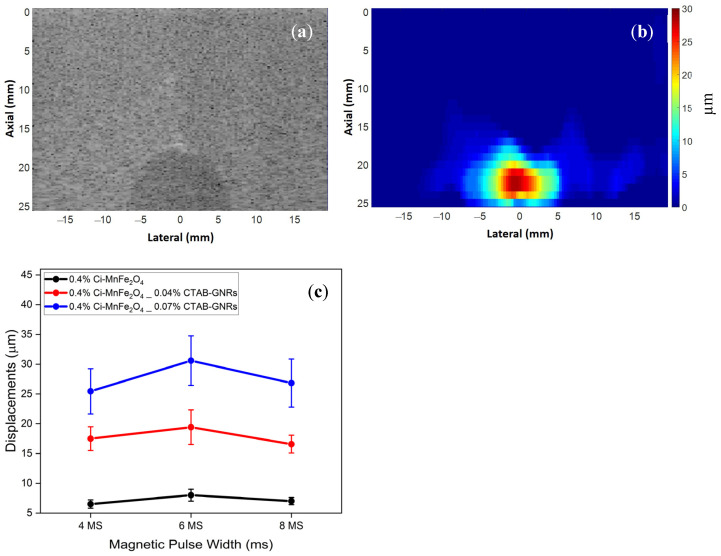
(**a**) The B-mode, (**b**) MMUS image of the phantom containing hybrid NPs of 0.4 wt% Ci-MnFe_2_O_4__0.07 wt% CTAB-GNRs, and (**c**) the induced displacements for phantoms containing Ci-MnFe_2_O_4_ and Ci-MnFe_2_O_4__CTAB-GNRs hybrid NPs.

The next step was to perform the MMUS measurements using the gelatin–agar phantoms containing inclusions labeled with Ci-MnFe_2_O_4_ (0.4 wt%) and hybrid NPs of Ci-MnFe_2_O_4_ (0.4 wt%)_CTAB-GNRs (0.04 wt% and 0.07 wt%). For example, a B-mode and an MMUS image of a phantom containing hybrid NPs of 0.4 wt% Ci-MnFe_2_O_4__0.07 wt% CTAB-GNRs are illustrated in Figure 8a,b, respectively. Figure 8b depicts the induced displacement of approximately 30 μm, displaying the inclusion region (where the NPs are located). Figure 8c shows the induced displacements for three phantoms using three different magnetic pulse widths with the same magnetic field amplitude. The induced displacement of a phantom labeled with 0.4 wt% Ci-MnFe_2_O_4__0.07 wt% CTAB-GNRs hybrid NPs was significantly greater at around 30.6 ± 4.16 μm than that of 0.4 wt% Ci-MnFe_2_O_4__0.04 wt% CTAB-GNRs (19.42 ± 2.9 μm) and the sample labeled with 0.4 wt% Ci-MnFe_2_O_4_ (8 ± 1 μm). Mehrmohammadi and Yoon et al. [62,63] also found that using small nanoclusters of MNPs with a size of 55 nm resulted in higher displacements for pulsed MMUS than using individual MNPs. Hence, a similar outcome was observed in our work by generating nanoclusters, which agrees with Mehrmohammadi and Yoon et al. [62,63].

**Figure 9:**
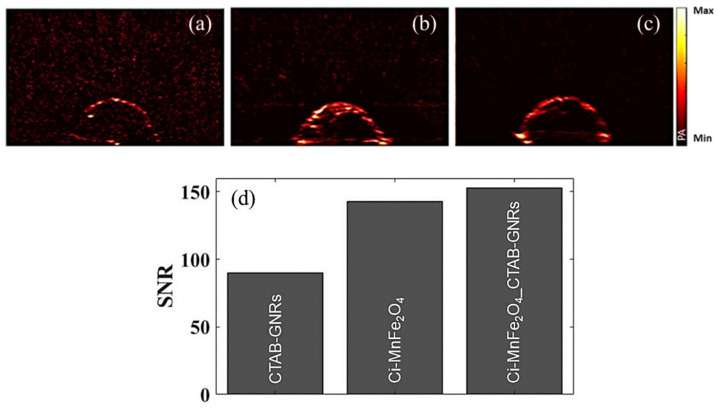
(**a**) PA images of the phantoms containing 0.04 wt% CTAB-GNRs, (**b**) 0.4 wt% Ci-MnFe_2_O_4_, and (**c**) 0.4 wt% Ci-MnFe_2_O_4__ 0.04 wt% CTAB-GNRs. The images cover a 25 mm by 40 mm area. (**d**) The SNR of PAI using different phantoms.

Furthermore, since GNRs have remarkable optical properties, these hybrid NPs were also preliminarily examined as PAI contrast agents. Figure 9a represents the PA image for the tissue-mimicking phantom only containing CTAB-GNRs (as an inclusion), and Figure 9b,c show the images of the phantoms containing Ci-MnFe_2_O_4_ and hybrid NPs (Ci-MnFe_2_O_4__CTAB-GNRs), respectively. Based on the results, although GNRs have been considered one of the most common metal NPs in PAI, the concentration used herein was very low (0.04 wt%); therefore, the obtained PA signal was not strong. The optical absorption for the next sample (only labeled with a high concentration of Ci-MnFe_2_O_4_) was boosted, which improved the image contrast, as shown in Figure 9b. The last sample (Figure 9c) containing the hybrid NPs also demonstrated a strong PA signal (like Figure 9b) since the number of particles increased by mixing Ci-MnFe_2_O_4_ and CTAB-GNRs. The signal-to-noise ratio (SNR) of the samples was also depicted in Figure 9d, and based on the results, the sample containing hybrid NPs reported a larger SNR (152.7) than that of Ci-MnFe_2_O_4_ (142.6) and GNRs (90.03). Therefore, these hybrid NPs may be applied as a plausible contrast agent for PAI.

**Figure 10:**
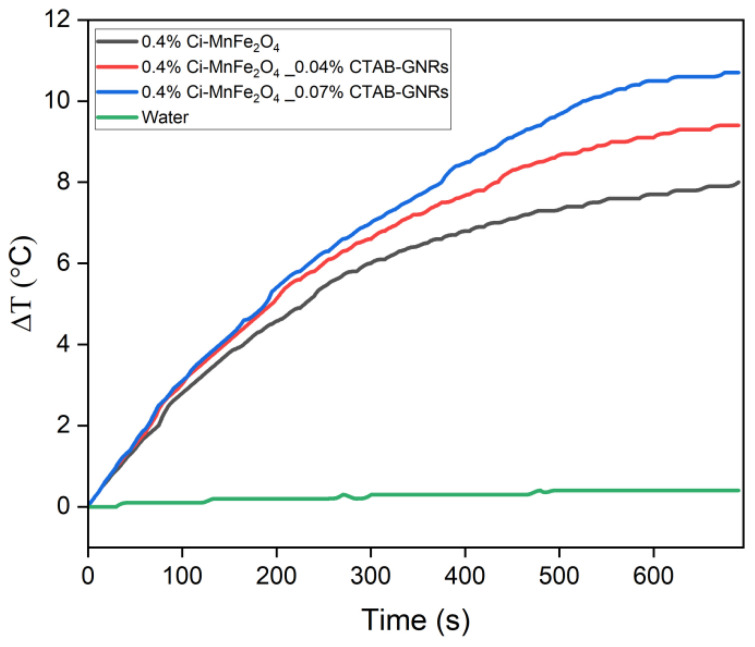
Temperature variation as a time function for Ci-MnFe_2_O_4_ and its combination with two different concentrations of CTAB-GNRs.

Additionally, the potential of both hybrid NPs of Ci-MnFe_2_O_4__CTAB-GNRs was also initially verified in MH as another application using the magnetic field with characteristics described in Section 2.7. Figure 10 shows the temperature variation as a function of time for all samples (0.4 wt% Ci-MnFe_2_O_4_ (sample 1), 0.4 wt% Ci-MnFe_2_O_4__0.04 wt% CTAB-GNRs (sample 2), 0.4 wt% Ci-MnFe_2_O_4__0.07 wt% CTAB-GNRs (sample 3)) and water (as a reference). SLP and ILP values of samples 1, 2, and 3 were 25.5 W/g, 3.02 nHm^2^ kg^−1^, 24.6 W/g, 2.9 nHm^2^ kg^−1^, and 23.8 W/g, 2.73 nHm^2^ kg^−1^, respectively. Due to the low concentration of IONPs used, the samples’ heating efficiency is less than 30 W/g. Although the SLP values, which are based on the initial slope of the heating curve, were almost similar for the three samples, the equilibrium temperature was higher for samples 2 and 3 (red and blue curves). One possible explanation is a better arrangement of magnetic anisotropy axes in the hybrid nanoclusters, possibly during AC field excitation. Brownian rotation helps orient the magnetic anisotropy axes of the MNPs, slightly enhancing the hyperthermia. Note that there is no contribution from the GNRs since the eddy’s current loss at this size is negligible. Further studies may help to evaluate if the GNRs are influencing the Néel collective relaxation of the aggregates of MnFe_2_O_4_ NPs coupled to the GNRs [27]. Nevertheless, these hybrid NPs may be used similarly to Ci-MnFe_2_O_4_ as a feasible heat generator for MH.

## 4. Conclusions

To conclude, hybrid NPs were prepared by a simple combination of positively charged GNRs coated with CTAB and negatively charged citrate-coated manganese ferrite. The electrostatic interaction of these NPs was studied using various characterizations, including UV-Visible, TEM, magnetic separation, and ATR. Interestingly, the higher the concentration of GNRs used, the greater the observed MMUS signal (induced displacement) from Ci-MnFe_2_O_4__CTAB-GNRs hybrid NPs. The cause was the formation of nanoclusters, which improved contrast in MMUS. In this regard, more research will be conducted as a next step to determine the optimal concentration of CTAB-GNRs to improve the contrast of MMUS in a subsequent study. Moreover, these potential candidates exhibited high stability and an absence of aggregation in the PBS medium. As a result, using the proposed multifunctional NPs for MMUS reduces the required dosage of NPs while potentially minimizing side effects [34]. Nonetheless, more research is needed to confirm this contrast improvement in MMUS before using these particles in vivo. It should also be noted that the concentration of CTAB-GNRs in the hybrid NPs was significantly lower than Ci-MnFe_2_O_4_. Furthermore, another advantage of these hybrid NPs is their potential for use in MH therapy and PAI. Hence, these hybrid NPs could be used simultaneously in imaging and thermal therapy due to their dual magnetic susceptibility and optical properties.

## Figures and Tables

**Table 1:** Zeta potential, hydrodynamic size, and PDI of CTAB-GNRs, Ci-MnFe_2_O_4_, and hybrid NPs in water. Hybrid NPs were also examined in a PBS medium.

Samples	Solution	Zeta Potential (mV)	Hydrodynamic Size (nm)	PDI
CTAB-GNRs	Water	41	-	-
Ci-MnFe_2_O_4_	Water	−43.5	38	0.32
0.4 wt% Ci-MnFe_2_O_4__0.04 wt% CTAB-GNRs	Water	−30.4	43	0.34
0.4 wt% Ci-MnFe_2_O_4__0.07 wt% CTAB-GNRs	Water	−31.1	37.5	0.33
0.4 wt% Ci-MnFe_2_O_4__0.04 wt% CTAB-GNRs	PBS	−37.3	38	0.4
0.4 wt% Ci-MnFe_2_O_4__0.07 wt% CTAB-GNRs	PBS	−33.8	44	0.4

## Data Availability

Not applicable.

## Supplementary Materials

The following supporting information can be downloaded at: https://www.mdpi.com/article/10.3390/nano13030434/s1, Figure S1: TEM images (the scale bars are 100 nm (a) and 200 nm (b)), and histogram of the particle size distribution of Ci-MnFe_2_O_4_ (c); Figure S2: The XRD patterns of Ci-MnFe_2_O_4_; Figure S3: Schematic preparation of gold seed (a) and GNRs (b); Figure S4: A Depiction of a pulsed magnetomotive ultrasound imaging system, which is mainly composed of an ultrasound acquisition setup integrated with a power pulse amplifier that drives the coil to generate the magnetic field excitation; Figure S5: The UV-Visible spectrum of the gold seed after 20 min (a) and its TEM image, (b) confirming the formation of small gold seeds; Figure S6: The UV-Visible absorption spectrum of CTAB-GNRs (a). The histogram of long-axis (length) (b) and short-axis (width) (c) of GNRs with an aspect ratio of 2.76.

## References

[1] Huang X., Neretina S., El-Sayed M.A. (2009). Gold nanorods: From synthesis and properties to biological and biomedical applications. Adv. Mater..

[2] Al-Eryani Y., Dadashi M., Aftabi S., Sattarifard H., Ghavami G., Oldham Z.W., Ghoorchian A., Ghavami S. (2021). Toxicity, therapeutic applicability, and safe handling of magnetic nanomaterials. Magnetic Nanomaterials in Analytical Chemistry.

[3] Arsalani S., Arsalani S., Hadadian Y., Sampaio D.R.T., Baffa O., Pavan T.Z., Carneiro A.A.O. (2019). The effect of magnetization of natural rubber latex-coated magnetite nanoparticles on shear wave dispersion magneto-motive ultrasound. Phys. Med. Biol..

[4] Durr N.J., Larson T., Smith D.K., Korgel B.A., Sokolov K., Ben-Yakar A. (2007). Two-photon luminescence imaging of cancer cells using molecularly targeted gold nanorods. Nano Lett..

[5] Nikoobakht B., El-Sayed M.A. (2003). Preparation and growth mechanism of gold nanorods (NRs) using seed-mediated growth method. Chem. Mater..

[6] Zhang L., Xia K., Bai Y.Y., Lu Z., Tang Y., Deng Y., Chen J., Qian W., Shen H., Zhang Z. (2014). Synthesis of gold nanorods and their functionalization with bovine serum albumin for optical Hyperthermia. J. Biomed. Nanotechnol..

[7] Chen Y.-S., Zhao Y., Yoon S.J., Gambhir S.S., Emelianov S. (2019). Miniature gold nanorods for photoacoustic molecular imaging in the second near-infrared optical window. Nat. Nanotechnol..

[8] Alkilany A.M., Nagaria P.K., Hexel C.R., Shaw T.J., Murphy C.J., Wyatt M.D. (2009). Cellular uptake and cytotoxicity of gold nanorods: Molecular origin of cytotoxicity and surface effects. Small.

[9] Arami H., Khandhar A., Liggitt D., Krishnan K.M. (2015). In vivo delivery, pharmacokinetics, biodistribution and toxicity of iron oxide nanoparticles. Chem. Soc. Rev..

[10] Arsalani S., Hadadian Y., Mazon E.E., Guidelli E.J., Kava E., Ramos A.P., Gualdi A.J., Pavan T.Z., Baffa O., Carneiro A.A.O. (2022). Uniform Size PEGylated Iron Oxide Nanoparticles as a Potential Theranostic Agent Synthesized by a Simple Optimized Coprecipitation Route. J. Magn. Magn. Mater.

[11] Arsalani S., Guidelli E.J., Silveira M.A., Salmon C.E.G., Araujo J.F.D.F., Bruno A.C., Baffa O. (2019). Magnetic Fe3O4 Nanoparticles Coated by Natural Rubber Latex as MRI Contrast Agent. J. Magn. Magn. Mater..

[12] Arsalani S., Radon P., Schier P., Jaufenthaler A., Liebl M., Baumgarten D., Wiekhorst F. (2022). Developing Magnetorelaxometry Imaging for Human Applications. Phys. Med. Biol..

[13] Arsalani S., Guidelli E.J., Araujo J.F.D.F., Bruno A.C., Baffa O. (2018). Green Synthesis and Surface Modification of Iron Oxide Nanoparticles with Enhanced Magnetization Using Natural Rubber Latex. ACS Sustain. Chem. Eng..

[14] Hadadian Y., Masoomi H., Dinari A., Ryu C., Hwang S., Kim S., Cho B.K., Lee J.Y., Yoon J. (2022). From Low to High Saturation Magnetization in Magnetite Nanoparticles: The Crucial Role of the Molar Ratios between the Chemicals. ACS Omega.

[15] Hadadian Y., Sampaio D.R.T., Ramos A.P., Carneiro A.A.O., Mozaffari M., Cabrelli L.C., Pavan T.Z. (2018). Synthesis and Characterization of Zinc Substituted Magnetite Nanoparticles and Their Application to Magneto-Motive Ultrasound Imaging. J. Magn. Magn. Mater.

[16] Arsalani S., Hadadian Y., Sampaio D.R.T., Arsalani S., Almedia T.W.J., Pavan T.Z., Carneiro A.A.O. (2019). A Study on Magnetic Nanoparticles Concentration in Shear Wave Dispersion Magnetomotive Ultrasound. IFMBE Proc..

[17] Gutierrez F.V., de Falco A., Yokoyama E., Mendoza L.A.F., Luz-Lima C., Perez G., Loreto R.P., Pottker W.E., la Porta F.A., Solorzano G. (2021). Magnetic Characterization by Scanning Microscopy of Functionalized Iron Oxide Nanoparticles. Nanomaterials.

[18] Próspero A.G., Quini C.C., Bakuzis A.F., Fidelis-de-Oliveira P., Moretto G.M., Mello F.P.F., Calabresi M.F.F., Matos R.V.R., Zandoná E.A., Zufelato N. (2017). Real-Time in Vivo Monitoring of Magnetic Nanoparticles in the Bloodstream by AC Biosuscep-tometry. J. Nanobiotechnology.

[19] Soares G.A., Prospero A.G., Calabresi M.F., Rodrigues D.S., Simoes L.G., Quini C.C., Matos R.R., Pinto L.A., Sousa-Junior A.A., Bakuzis A.F. (2019). Multichannel AC Biosusceptometry System to Map Biodistribution and Assess the Pharmacokinetic Profile of Magnetic Nanoparticles by Imaging. IEEE Trans. Nanobioscience.

[20] Prospero A.G., Buranello L.P., Fernandes C.A.H., dos Santos L.D., Soares G., C Rossini B., Zufelato N., Bakuzis A.F., de Mattos Fontes M.R., de Arruda Miranda J.R. (2021). Corona Protein Impacts on Alternating Current Biosusceptometry Signal and Circulation Times of Differently Coated MnFe2O4 Nanoparticles. Nanomedicine.

[21] Quini C.C., Matos J.F., Próspero A.G., Calabresi M.F.F., Zufelato N., Bakuzis A.F., Baffa O., Miranda J.R.A. (2015). Renal Perfusion Evaluation by Alternating Current Biosusceptometry of Magnetic Nanoparticles. J. Magn. Magn. Mater.

[22] Nunes A.D.C., Ramalho L.S., Souza Á.P.S., Mendes E.P., Colugnati D.B., Zufelato N., Sousa M.H., Bakuzis A.F., Castro C.H. (2014). Manganese Ferrite-Based Nanoparticles Induce Ex Vivo, but Not in Vivo, Cardiovascular Effects. Int. J. Nanomed..

[23] Nunes A.D.C., Gomes-Silva L.A., Zufelato N., Prospero A.G., Quini C.C., Matos R.V.R., Miranda J.R.A., Bakuzis A.F., Castro C.H. (2019). Albumin Coating Prevents Cardiac Effect of the Magnetic Nanoparticles. IEEE Trans. Nanobioscience.

[24] Yang Y., Shi H., Wang Y., Shi B., Guo L., Wu D., Yang S., Wu H. (2015). Graphene Oxide/Manganese Ferrite Nanohybrids for Magnetic Resonance Imaging, Photothermal Therapy and Drug Delivery. J. Biomater. Appl..

[25] Islam K., Haque M., Kumar A., Hoq A., Hyder F., Hoque S.M. (2020). Manganese Ferrite Nanoparticles (MnFe2O4): Size Dependence for Hyperthermia and Negative/Positive Contrast Enhancement in MRI. Nanomaterials.

[26] Branquinho L.C., Carrião M.S., Costa A.S., Zufelato N., Sousa M.H., Miotto R., Ivkov R., Bakuzis A.F. (2013). Effect of magnetic dipolar interactions on nanoparticle heating efficiency: Implications for cancer hyperthermia. Sci. Rep..

[27] Zufelato N., Aquino V.R.R., Shrivastava N., Mendanha S., Miotto R., Bakuzis A.F. (2022). Heat Generation in Magnetic Hyperthermia by Manganese Ferrite-Based Nanoparticles Arises from Néel Collective Magnetic Relaxation. ACS Appl. Nano Mater..

[28] Verde E.L., Landi G.T., Carrião M.S., Drummond A.L., Gomes J.A., Vieira E.D., Sousa M.H., Bakuzis A.F. (2012). Field dependent transition to the non-linear regime in magnetic hyperthermia experiments: Comparison between maghemite, copper, zinc, nickel and cobalt ferrite nanoparticles of similar sizes. AIP Adv..

[29] Mehrmohammadi M., Homan K., Joshi P., Emelianov S., Chen Y.-S., Sokolov K., Mallidi S., Truby R., Qu M. (2011). Magne-to-Photo-Acoustic Imaging. Biomed. Opt. Express.

[30] Qu M., Mallidi S., Mehrmohammadi M., Ma L.L., Johnston K.P., Sokolov K., Emelianov S. (2009). Combined Photoacoustic and Magneto-Acoustic Imaging. Conf. Proc. IEEE Eng. Med. Biol. Soc..

[31] Qu M., Mehrmohammadi M., Truby R., Graf I., Homan K., Emelianov S. (2014). Contrast-Enhanced Magneto-Photo-Acoustic Imaging in Vivo Using Dual-Contrast Nanoparticles. Photoacoustics.

[32] Urries I., Muñoz C., Gomez L., Marquina C., Sebastian V., Arruebo M., Santamaria J. (2014). Magneto-Plasmonic Nanoparticles as Theranostic Platforms for Magnetic Resonance Imaging, Drug Delivery and NIR Hyperthermia Applications. Nanoscale.

[33] Qu M., Mehrmohammadi M., Emelianov S. (2011). Detection of nanoparticle endocytosis using magneto-photoacoustic imaging. Small.

[34] Mehrmohammadi M., Ma L.L., Chen Y.-S., Qu M., Joshi P., Chen R.M., Johnston K.P., Emelianov S. (2010). Combined Photothermal Therapy and Magneto-Motive Ultrasound Imaging Using Multifunctional Nanoparticles. Nanoscale Imaging Sensing and Actuation for Biomedical Applications VII.

[35] Morasso C., Picciolini S., Schiumarini D., Mehn D., Ojea-Jiménez I., Zanchetta G., Vanna R., Bedoni M., Prosperi D., Gramatica F. (2015). Control of Size and Aspect Ratio in Hydroquinone-Based Synthesis of Gold Nanorods. J. Nanoparticle Res..

[36] Arsalani S., Löwa N., Kosch O., Radon P., Baffa O., Wiekhorst F. (2021). Magnetic Separation of Iron Oxide Nanoparticles to Improve Their Application for Magnetic Particle Imaging. Phys. Med. Biol..

[37] Leong S.S., Ahmad Z., Lim J. (2015). Magnetophoresis of superparamagnetic nanoparticles at low field gradient: Hydrodynamic effect. Soft Matter.

[38] Andreu J., Camacho J., Faraudo J., Benelmekki M., Rebollo C., Martínez L.M. (2011). Simple analytical model for the magnetophoretic separation of superparamagnetic dispersions in a uniform magnetic gradient. Phys. Rev. E.

[39] Leong S.S., Yeap S.P., Lim J.K. (2016). Working principle and application of magnetic separation for biomedical diagnostic at high-and low-field gradients. Interface Focus.

[40] De Las Cuevas. G., Faraudo J., Camacho J. (2008). Low-gradient magnetophoresis through field-induced reversible aggregation. J. Phys. Chem. C.

[41] Lim J., Lanni C., Evarts E.R., Lanni F., Tilton R.D., Majetich S.A. (2011). Magnetophoresis of nanoparticles. ACS Nano.

[42] Leong S.S., Ahmad Z., Low S.C., Camacho J., Faraudo J., Lim J.K. (2020). Unified view of magnetic nanoparticle separation under magnetophoresis. Langmuir.

[43] Pavan T.Z., Madsen E.L., Frank G.R., Adilton O Carneiro A., Hall T.J. (2010). Nonlinear elastic behavior of phantom materials for elastography. Phys. Med. Biol.

[44] Mazon E.E., Arsalani S., Uliana J.H., Carneiro A.A.O., Gualdi A.J., Pavan T.Z. A Pulsed Magnetomotive Ultrasound Imaging System for Magnetic Nanoparticle Detection. Proceedings of the 2021 IEEE UFFC Latin America Ultrasonics Symposium (LAUS).

[45] Sampaio D.R.T., Grillo F.W., Bruno A.C., Pavan T.Z., Carneiro A.A.O. (2017). A magneto-motive ultrasound platform designed for pre-clinical and clinical applications. Res. Biomed. Eng..

[46] Uliana J.H., Sampaio D.R.T., Fernandes G.S.P., Brassesco M.S., Nogueira-Barbosa M.H., Carneiro A.A.O., Pavan T.Z. (2020). Multiangle long-axis lateral illumination photoacoustic imaging using linear array transducer. Sensors.

[47] Hadadian Y., Azimbagirad M., Navas E.A., Pavan T.Z. (2019). A versatile induction heating system for magnetic hyperthermia studies under different experimental conditions. Rev. Sci. Instrum..

[48] Patil R., Thorat N.D., Shete P.B., Otari S.V., Tiwale B.M., Pawar S.H. (2016). In vitro hyperthermia with improved colloidal stability and enhanced SAR of magnetic core/shell nanostructures. Mater. Sci. Eng..

[49] Hadadian Y., Ramos A.P., Pavan T.Z. (2019). Role of zinc substitution in magnetic hyperthermia properties of magnetite nanoparticles: Interplay between intrinsic properties and dipolar interactions. Sci. Rep..

[50] Hadadian Y., Uliana J.H., Carneiro A.A.O., Pavan T.Z. (2020). A novel theranostic platform: Integration of magnetomotive and thermal ultrasound imaging with magnetic hyperthermia. IEEE Trans. Biomed. Eng..

[51] Mazon E., Villa-Martínez E., Hernández-Sámano A., Córdova-Fraga T., Ibarra-Sánchez J.J., Calleja H.A., Leyva Cruz J.A., Barrera A., Estrada J.C., Paz J.A. (2017). A high-resolution frequency variable experimental setup for studying ferrofluids used in magnetic hyperthermia. Rev. Sci. Instrum..

[52] Kumbhar A.S., Kinnan M.K., Chumanov G. (2005). Multipole plasmon resonances of submicron silver particles. J. Am. Chem. Soc..

[53] Guidelli E.J., Araujo L.F., Assunção A.C.A., Carvalho I.C.S., Clarke D.R., Baffa O. (2020). Microwave-Assisted Growth of Silver Nanoparticle Films with Tunable Plasmon Properties and Asymmetrical Particle Geometry for Applications as Radiation Sensors. Plasmonics.

[54] Amendola V., Bakr O.M., Stellacci F. (2010). A study of the surface plasmon resonance of silver nanoparticles by the discrete dipole approximation method: Effect of shape, size, structure, and assembly. Plasmonics.

[55] Truby R., Emelianov S., Homan K.A. (2013). Ligand-mediated self-assembly of hybrid plasmonic and superparamagnetic nanostructures. Langmuir.

[56] Bakuzis A.F., Branquinho L.C., Luiz E Castro L., de Amaral E Eloi M.T., Miotto R. (2013). Chain formation and aging process in biocompatible polydisperse ferrofluids: Experimental investigation and Monte Carlo simulations. Adv. Colloid Interface Sci..

[57] Shaw D.J. (1980). Introduction to Colloid and Surface Chemistry.

[58] Chen Z.P., Zhang Y., Xu K., Xu R.Z., Uu J.W., Gu N. (2008). Stability of hydrophilic magnetic nanoparticles under biologically relevant conditions. J. Nanosci. Nanotechnol..

[59] Wan J., Wang J.H., Liu T., Xie Z., Yu X.F., Li W. (2015). Surface chemistry but not aspect ratio mediates the biological toxicity of gold nanorods in vitro and in vivo. Sci. Rep..

[60] Cao Q., Zhao H., He Y., Li X., Zeng L., Ding N., Wang J., Yang J., Wang G. (2010). Hydrogen-bonding-induced colorimetric detection of melamine by nonaggregation-based Au-NPs as a probe. Biosens. Bioelectron..

[61] Wittmann L., Turrina C., Schwaminger S. (2021). The Effect of pH and Viscosity on Magnetophoretic Separation of Iron Oxide Nanoparticles. Magnetochemistry.

[62] Mehrmohammadi M., Yoon K.Y., Qu M., Johnston K.P., Emelianov S.Y. (2010). Enhanced pulsed magneto-motive ultrasound imaging using superparamagnetic nanoclusters. Nanotechnology.

[63] Yoon K.Y., Mehrmohammadi M., Borwankar A., Emelianov S.Y., Johnston K.P. (2015). Synthesis of Iron Oxide Nanoclusters with Enhanced Magnetization and Their Applications in Pulsed Magneto-Motive Ultrasound Imaging. Nano.

