# Supplementary material for: Hybrid Nanoparticles of Citrate-Coated Manganese Ferrite and Gold Nanorods in Magneto-Optical Imaging and Thermal Therapy
Source: Nanomaterials (Basel). 2023 Jan 20;13(3):434. doi: 10.3390/nano13030434 (PMC9921964; doi:10.3390/nano13030434)
Supplement: Supplementary file 1 [file nanomaterials-13-00434-s001.zip › nanomaterials-2122620-supplementary.pdf]

nanomaterials

Supporting Materials

Hybrid Nanoparticles of Citrate-Coated Manganese Ferrite and Gold Nanorods in Magneto-optical Imaging and Thermal Therapy

Saeideh Arsalani [1], Soudabeh Arsalani [2], Mileni Isikawa [1], Eder Jose Guidelli [1], Ernesto E. Mazon [1], Ana Paula Ramos [3], Andris Bakuzis [4], Theo Z. Pavan [1], Oswaldo Baffa [1] and Antonio A.O. Carneiro[1,] *

[1] Department of Physics, FFCLRP, University of São Paulo, Av. Bandeirantes 3900, Ribeirão Preto 14040-901, São Paulo, Brazil; arsalani@usp.br (S.A.); mileni.isikawa@usp.br (M.I.); guidelli@usp.br (E.J.G.); ernestomazon@usp.br (E.E.M.); theozp@usp.br (T.Z.P.); baffa@usp.br (O.B.)
[2] Physikalisch-Technische Bundesanstalt, Abbestrasse 2-12, D-10587 Berlin, Germany; soudabeh.arsalani@ptb.de
[3] Department of Chemistry, FFCLRP, University of São Paulo, Av. Bandeirantes 3900, Ribeirão Preto 14040-901, São Paulo, Brazil; anapr@ffclrp.usp.br
[4] Institute of Physics and CNanoMed, Federal University of Goiás, Goiânia, GO 74690 900,Brazil; bakuzis@ufg.br
* Correspondence: adilton@usp.br; Tel.: +55-16991980030

The MnF_2O_4 was synthesized via co-precipitation method by mixing $MnCl_2.4H_2O$ and $FeCl_4.6H_2O$ in 1:2 molar ratios, and adding to basic solutions (CH_3NH_2), then heating to form $MnFe_2O_4$. Following that, sodium citrate was added to the synthesized MNPs at 80 °C and stirred for a while to allow the surfactant to act. A detailed description of the synthesis of these MNPs can be found in Zufelato et al.[27].The morphology and size distribution of Ci-$MnFe_2O_4$ were determined by TEM. The TEM images of manganese ferrite capped with sodium citrate showed spherical morphology (Figure S1a, b) with mean particle diameters of 16.7 ± 4.8 nm (Figure S1c) and PDI of 0.32.

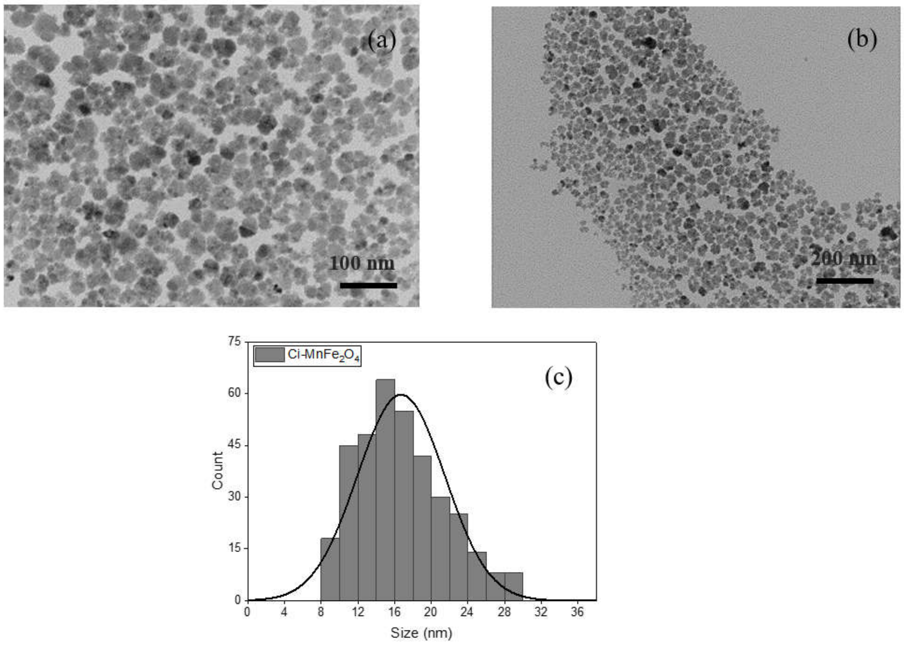

Figure S1. TEM images (the scale bars are 100 nm (a) and 200 nm (b)), and histogram of the particle size distribution of Ci-$MnFe_2O_4$ (c).

Figure S2. provides information about the composition and size of Ci-$MnFe_2O_4$ by an XRD measurement. The data confirmed the spinal structure of ferrite and reported the average crystallite size of 14 nm using the Scherrer equation. The estimated crystallite size is in good agreement with TEM results, indicating that each particle consists of a crystallite.

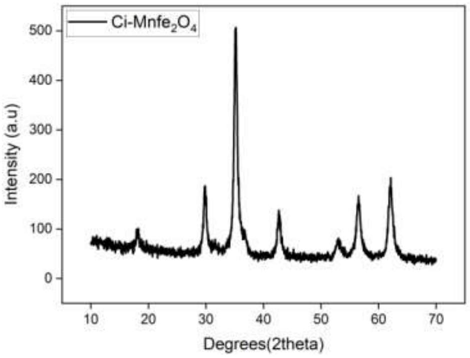

Figure S2. The XRD patterns of Ci-$MnFe_2O_4$.

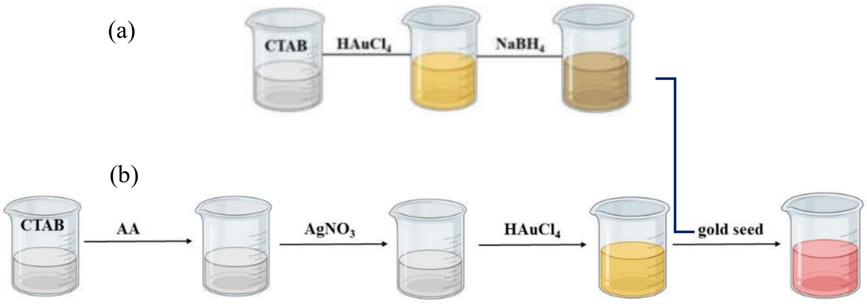

Figure S3. Schematic preparation of gold seed (a) and GNRs (b).

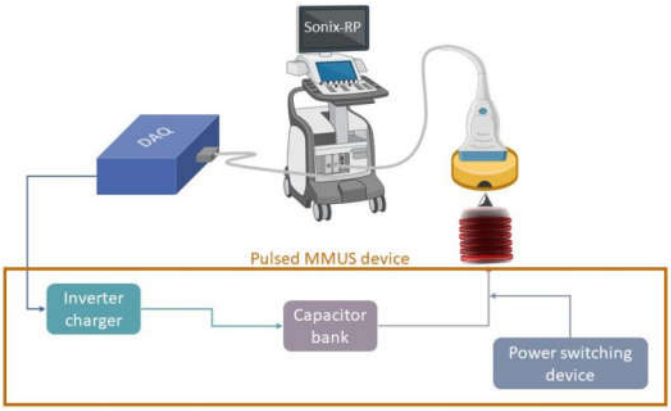

Figure S4. A Depiction of a pulsed magnetomotive ultrasound imaging system, which is mainly composed of an ultrasound acquisition setup integrated with a power pulse amplifier that drives the coil to generate the magnetic field excitation.

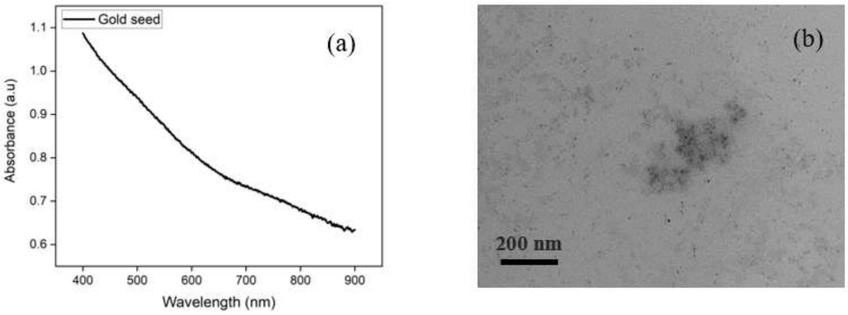

Figure S5. The UV-Visible spectrum of the gold seed after 20 minutes (a) and its TEM image (b) confirming the formation of small gold seeds.

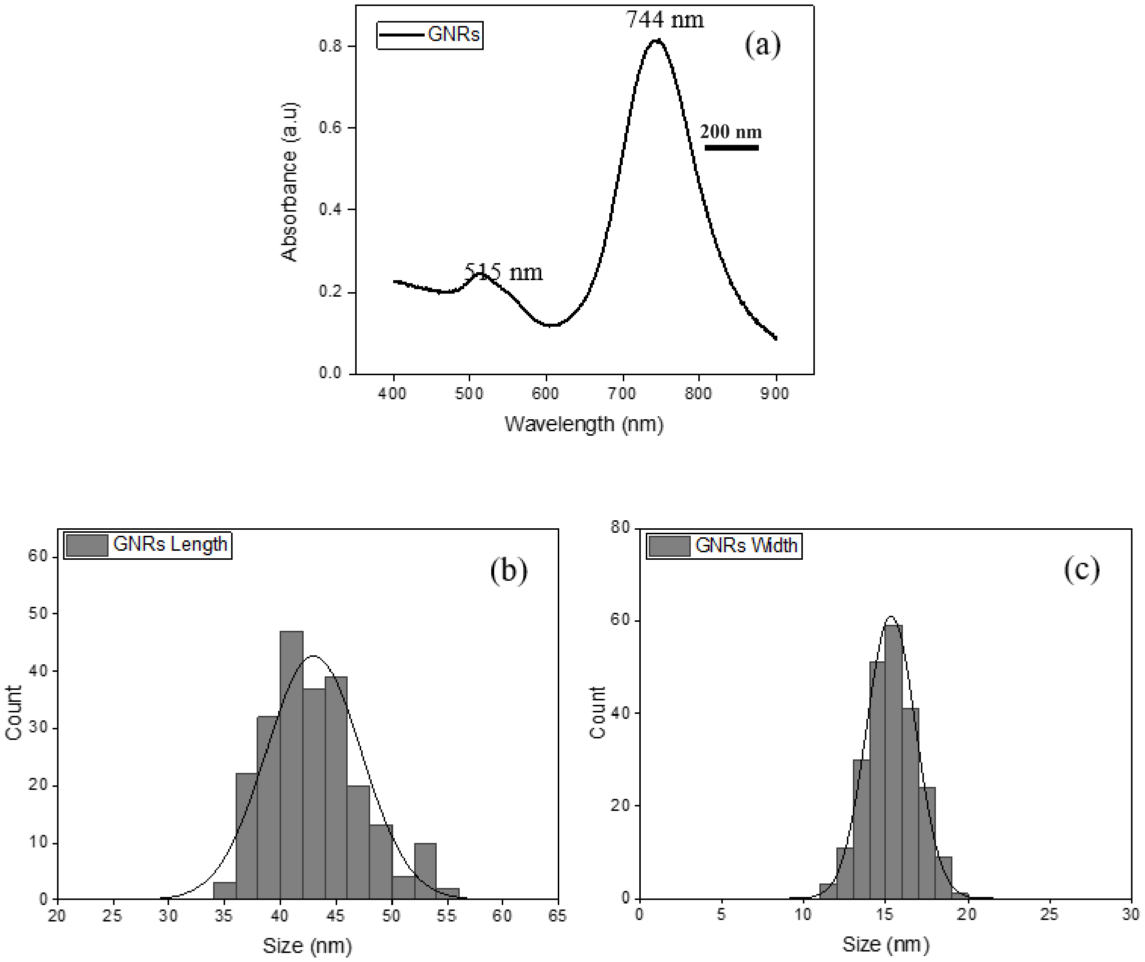

Figure S6. The UV-Visible absorption spectrum of GNRs (a). The histogram of long-axis (length) (b) and short-axis (width) (c) of GNRs with an aspect ratio of 2.76.

Reference

27. Zufelato, N., et al., Heat Generation in Magnetic Hyperthermia by Manganese Ferrite-Based Nanoparticles Arises from Néel Collective Magnetic Relaxation. ACS Applied Nano Materials, 2022. **5**(5): p. 7521-7539.
